# The Surgical Anatomy of the Lymphatic Glands

**Published:** 1899-12

**Authors:** 


**The Surgical Anatomy of the Lymphatic Glands.** By Cecil H. Leaf, M.B. Pp. 72. Westminster [London]: Archibald Constable & Co. 1898.

The object of the author has been to indicate, by a series of diagrams with descriptive text, the position of the main groups of lymphatic glands in the human body, and to show the important bearing of this in surgical practice.

In his introductory remarks he draws attention to the great value of passing an excessive quantity of formalin solution (one in seven of water) under a very high pressure into the cadaver, to prepare it for the dissection of the lymphatic vessels. The solution escaping into the tissues hardens and dries up the fat in which the lymphatic vessels are embedded, and also hardens the walls of the vessels themselves, rendering their subsequent dissection easy. Using this method, Mr. Leaf demonstrated in one instance an afferent lymphatic vessel partly terminating in an inguinal gland and partly, by a branch, in a vein; and in another instance an efferent lymphatic from an inguinal gland terminated directly in a vein. These observations are of great interest, and it is important to determine if such communications between lymphatics and veins are common throughout the body.

The positions of the lymphatic glands, in all parts, are clearly shown in eighteen full-page illustrations. These are coloured, and from the point of view of clearness leave nothing to be desired. In the text the author gives a concise description of the position of the glands, taking them in groups.

The book well fulfils the aim of its author, and we cordially recommend its study to those interested in this branch of anatomy. We should like to see in another edition some of the blemishes removed, which are either the fault of the proof reader or the result of a slip-shod style. Instances are to be seen in the sixth line of page 25, in line five of page 30, and in line fourteen of page 41.

